# Prednisolone treatment in acute interstitial nephritis (PRAISE) – protocol for the randomized controlled trial

**DOI:** 10.1186/s12882-021-02372-4

**Published:** 2021-05-01

**Authors:** Frank H. Mose, Henrik Birn, Nikolai Hoffmann-Petersen, Jesper N. Bech

**Affiliations:** 1 University Clinic in Nephrology and Hypertension, Aarhus University and Gødstrup Hospital, Herning, Denmark; 2 Department of Medicine, Gødstrup Hospital, Herning, Denmark; 3 Department of Renal Medicine, Aarhus University Hospital, Aarhus, Denmark; 4 Department of Biomedicine, Aarhus University, Aarhus, Denmark

**Keywords:** Acute interstitial nephritis, Prednisolone, Renal failure

## Abstract

**Background:**

Acute interstitial nephritis (AIN) is an important and common cause of acute renal failure. There are no generally accepted guidelines for the treatment of AIN, due to the lack of prospective randomized trials. Since AIN is characterized by an enhanced immune response, immunosuppressive treatment could potentially improve prognosis by attenuating inflammation and subsequent fibrosis. Despite the limited evidence of effects of steroids and potential adverse effects, prednisolone is frequently used in the treatment of AIN and there is a strong need for clinical trials on the effects of immunosuppression, including steroids, in the treatment of AIN. We aimed to evaluate the effectiveness of prednisolone treatment in AIN, and hypothesized a positive effect of prednisolone treatment on renal function in AIN.

**Methods:**

The study is a randomized, controlled, prospective, open label multicenter study, including incident adult patients with biopsy proven AIN. Patients will be randomized 1:1 to one of 2 treatment regimens:

A. No prednisolone treatment (control group) and
B. B) Oral prednisolone treatment staring with 60 mg daily tapered over 8 weeks.

One hundred ten patients (55 in each group) are planned to be included and followed for 1 year. Primary outcome is renal function estimated by eGFR 3 months after inclusion. Secondary outcomes are renal function after 12 months and need for renal replacement therapy and quality of life after 3 and 12 months. In addition, with-in prednisolone group analysis are performed to estimate the importance of treatment delay. Exploratory analyses include analysis of biomarkers in urine and plasma and the evaluation of these biomarkers in relation to renal prognosis and re-evaluation of renal biopsies to identify possible renal prognostic factors.

**Discussion:**

Strengths and possible limitations in the design are evaluated. The study will provide important information on the effects of prednisolone treatment in AIN and as well as prognostic information relevant for future use of biomarkers and histology. Ultimately, this would lead to improved and evidence based clinical guidelines for the treatment of AIN.

**Trial registration:**

ClinicalTrials.gov identifier NCT04376216 (Retrospectively registered on May 6, 2020).

## Background

Acute renal failure is associated with greatly increased mortality and morbidity irrespective of its cause [1, 2]. Acute interstitial nephritis (AIN) is an important and common cause of acute renal failure and occurs in 13–27% of renal biopsies performed in acute renal failure requiring dialysis in 40% of cases [3–6]. Histologically AIN is characterized by peritubular inflammation while glomeruli and vessels are typically spared [7]. Although some patients recover renal function completely, as many as 30–70% do not fully regain renal function leading to chronic kidney disease (CKD) [6, 8–14]. CKD is associated with increased morbidity and mortality. Two recent studies suggested that the risk of end stage renal disease requiring dialysis after biopsy proven AIN is 5–7% [6, 13].

In addition to renal failure AIN may present with joint pain (45%), fever (36%), skin rash (22%), eosinophilia (35%), microscopic hematuria (67%), macroscopic hematuria (5%), leukocyturia (82%), non-nephrotic proteinuria (93%), nephrotic proteinuria (3%) and nephrotic syndrome (1%) [15]. AIN can be subdivided in 3 categories based on the precipitating cause: drug induced (75%), infection (5–10%) or autoimmune diseases (15–20%) [5, 7, 16–18]. The pathophysiological mechanisms leading to renal inflammation in AIN are not fully clarified. Fibrotic changes can be observed 7–10 days after the beginning of the inflammatory process and if the triggering cause is not eliminated, the inflammation may proceed to interstitial fibrosis, tubular atrophy and chronic renal failure [19]. Studies have shown that macrophages, lymphocytes and activated tubular cells produce various cytokines that stimulate proliferation of fibroblast cells leading to fibrosis [20, 21]. Transforming growth factor-β1 (TGF-β1) promotes fibroblast activity in vitro and alters the phenotype of mesangial cells to a more fibroblast-like phenotype [22, 23]. Transgenetic mice overesxpressing TGF-β1 show increased renal fibrosis [24, 25]. Thus, TGF-β1 appears to play a central role in the development of renal fibrosis [26]. Bone morphogenetic protein-7 (BMP-7), on the other hand is a natural antagonist of TGF-β1 with anti-fibrotic and anti-inflammatory properties [26]. The roles of TGF-β1 and BMP-7 in AIN; however, have not been elucidated.

There are no generally accepted guidelines for the treatment of AIN [27], but normally this would include elimination of the precipitating cause and stimulus leading to renal inflammation. Since AIN is characterized by an enhanced immune response, immunosuppressive treatment could potentially improve prognosis by attenuating inflammation and subsequent fibrosis. Case series have suggested positive effects of steroid treatment, but only few larger, retrospective registry based studies have examined this [6, 8, 9, 12–14, 19, 28]. At present, only three retrospective studies with more than 100 patients have been published [29–31]. Muriithi and colleagues identified 133 cases with AIN, of which 95 were drug-induced [31, 32]. The study showed no difference in renal function after 1, 3 and 6 months when comparing 83 steroid-treated with 12 non-steroid-treated patients. In steroid-treated patients, a longer time from the onset of symptoms or biopsy to the initiation of steroid treatment was associated with inferior restoration of renal function. A retrospective, Scottish study including 171 cases of drug-induced AIN also failed to demonstrate an effect of steroid treatment on renal function after 12 months of follow-up [30]. As in the previous study, the steroid-treated (*n* = 124) had higher baseline creatinine levels. In contrast, a recent study from England including 187 patients showed a possible positive effect of steroid treatment [29]. After 24 months, eGFR was 42 ml/min in the steroid-treated group versus 24 ml/min in non-steroid-treated patients. In addition, there was a tendency towards fewer patients requiring of dialysis after 24 months among those treated with steroid. Baseline renal function was comparable among the steroid- and non-steroid-treated patients. Interestingly, a sub-analysis including only patients with drug-triggered AIN, i.e. excluding patients with autoimmune disease, did not show any significant effect of steroid treatment. This indicates that steroid treatment may only benefit patients with autoimmune related AIN. Retrospective studies are hampered by selection bias and there are no published, prospective, randomized studies evaluating the effects of steroids in AIN leaving the use of steroids in AIN controversial. Steroid treatment has several physical and mental adverse effects, such as diabetes mellitus, hypertension, cataract, osteoporosis and manic episodes and is associated with hyperlipidemia, obesity and osteonecrosis [33]. Despite the limited evidence of effects of steroids and potential adverse effects, prednisolone is frequently used in the treatment of AIN and there is a strong need for clinical trials on the effects of immunosuppression, including steroids, in the treatment of AIN.

The usefulness of biomarkers to diagnose and to define prognostic and therapeutic subgroups in AIN has not been extensively studied [34, 35]. Potentially useful biomarkers in plasma or urine include neutrophil gelatinase-associated lipocalin (NGAL), N-acetyl-beta-D-glucosaminidase (NAG), kidney injury marker 1 (KIM-1), tissue inhibitor of metalloproteinases-2 (TIMP-2), insulin-like growth factor-binding protein 7 (IGFBP7), interleukin 18 (IL-18) and monocyte chemotactic peptide-1 (MCP-1) [36–40]. In particular, Urinary NGAL excretion may be helpful for the diagnosis and to establish prognosis, while urinary MCP-1 excretion has been correlated with the degree of renal inflammation and edema in drug-induced AIN [40]. Alpha-1-microgolbulin (A1M) and Beta-2-macroglobulin (B2M) are low molecular weight proteins that are freely filtered in the glomeruli and reabsorbed in the proximal tubule [18]. Tubular damage leads to increased urinary excretion and both A1M and B2M may used to assess proximal tubule damage. So far these have been mostly studied in chronic hereditary forms of interstitial nephritis, such as Balkan nephropathy and tubulointerstitial nephritis with uveitis (TINU) [41–44]. Thus, further studies are needed to establish the potential value of biomarkers for the diagnosis and management of AIN.

### Hypotheses

This study will examine the following hypotheses:

1. Prednisolone treatment in AIN is associated with a higher eGFR 3 and 12 months after inclusion when compared to no prednisolone treatment.
2. Prednisolone treatment in AIN is associated with a greater decline in plasma creatinine from inclusion to 3 and 12 months after when compared to no prednisolone treatment.
3. Prednisolone treatment in AIN shortens the time to remission or partial remission when compared to no prednisolone treatment.
4. Prednisolone treatment in AIN reduces the number of patients requiring renal replacement therapy when compared to no prednisolone treatment.
5. A greater timespan from the presentation of AIN to the initiation of prednisolone treatment is associated with a lower eGFR 3 and 12 months after inclusion.

### Design

An investigator initiated, randomized, controlled, prospective, open label multicenter study, including incident adult patients with biopsy proven AIN. Patients will be randomized 1:1 to one of 2 treatment regimens:

A. No prednisolone treatment (control group)
B. Oral prednisolone treatment tapered over 8 weeks.

### Trial subjects

#### Recruitment

Patients are recruited consecutively at all Danish nephrology departments by local investigators.

#### Inclusion criteria

- Clinical suspicion of AIN
- Renal biopsy consistent with AIN
- Age ≥ 18 years
- One of following criteria:
  - ○ Plasma creatinine > 120 μmol/L or
  - ○ Plasma creatinine increase > 30 μmol/L or > 50% when compared to baseline plasma creatinine
- Informed consent

#### Exclusion criteria

- Inability to give informed consent
- Immunosuppressive treatment (including prednisolone) within 3 months prior to biopsy
- Any known systemic autoimmune disease
- Prednisolone intolerance
- Pregnancy or lactation
- Active cancer (except for basal cell carcinoma)
- Short life expectancy (< 6 months)
- CKD stage IV-V
- AIN secondary to or accompanied by glomerulonephritis, sarcoidosis or inherited interstitial renal disease
- Previous participation in this study

#### Withdrawal criteria

- Development of any exclusion criterion
- Withdrawal of consent

#### Screening

All patients with a renal biopsy consistent with AIN are screened for participation and included in the screening log.

#### Randomization

Randomization is performed locally and electronically using REDCap (www.redcap.au.dk; Clinical Trial Unit, Aarhus University). Randomization is performed 1:1 in blocks of 6. Since only a few subjects are expected to be included each center, any center effects are considered insignificant. Neither clinicians nor patients are blinded to the study treatment group.

### Study overview

Overview of the study from inclusion to week 52 is shown in Table 1.

**Table 1:** Overview of the study from inclusion to week 52

		Day 0^a^	Day 2	Day 4	Week 1	Week 2	Week 4	Week 8	Week 12	Week 26	Week 52
Treatment	A	No treatment									
	B	Oral prednisolone tapered over 8 weeks									
Medical history		+									
Biochemistry A		+	+	+	+	+	+	+	+	+	+
Biochemistry B		+							+	+	+
Biochemistry C		+			+		+		+		+
Study medication (group B)		+			+	+	+	+			
Blood pressure, HR		+	+	+	+	+	+	+	+	+	+
Clinical examination		+			+	+	+	+	+	+	+
24 h urine		+					+		+		+
Spot urine A		+	+	+	+	+	+	+	+	+	+
Spot urine B		+			+		+		+		+
Questionnaire (SF36)		+					+		+		+

Biochemistry A: P-sodium, p-potassium, p-total calcium, p-ionized calcium, p-phosphate, p-creatinine, eGFR, p-urea, p-glucose, WBC, p-c-reactive protein (CRP), b-sedimentation rate, p-PTH

Biochemistry B: Hemoglobin A1C. p-TSH, p-25-Hydroxy-Vitamine D2 + D3

Biochemistry C: TGF-β1, BMP-7

Study medication: Dose and adverse effects

Clinical examination: Body weight and physical examination

24-h urine collection: Urinary volume and urinary sodium, creatinine, and albumin excretion

Spot urine A: Urine-dip stick (hemoglobin, protein, leucocytes, glucose, nitrite), urinary albumin and creatinine concentrations and urinary albumin/creatinine ratio

Spot urine B: Urinary concentration and ratios in relation to urinary creatinine of TGF-β1, BMP-7, A1M, B2M, MCP-1, NGAL, NAG, IL-18, TIMP-2, KIM-1, IGFBP7 and CD163-

Questionnaire: SF36 is filled in by the patient

^a^Before start of study medication

### Study treatment

Subject are randomized to one of two treatments:

- Group A (controls): No trial medication
- Group B: Oral prednisolone for 8 weeks with dose tapering (Table 2)

**Table 2:** Prednisolone dosages and tapering over 8 weeks after inclusion

Prednisolone dosing regime		
Day	Week	Dose (mg/day)
1–14	1–2	60
15–28	3–4	40
29–35	5	30
36–42	6	20
43–49	7	10
50–56	8	5

Deviations from the dose-tapering regimen are tolerated if considered of vital clinical importance as per discretion of the local investigator. Such deviations are recorded in the electronic case report form (eCRF).

Monitoring of compliance with prednisolone is performed at study visits by interview, as well as by counting the returned study medication after cession of intake.

Prednisolone is used extensively in nephrology patients, and most nephrologists are familiar with the drug. Prednisolone is administered to the patients with-out costs. Side effects are expected in most cases. Adverse reactions will be registered in eCRFs at all planned clinical visits and from the questionnaires.

#### Other treatment

Patients allocated to prednisolone are advised to take oral vitamin D3, 20 μg daily. Furthermore, a proton pump inhibitor is recommended while the dose of prednisolone exceeds 15 mg/day, unless this is considered the precipitating cause of AIN. Bisphofonates are not recommended; however, patients already taken these at inclusion will continue.

No other immunosuppressive treatment may be prescribed during the study; however, other additional treatment is allowed as per local guidelines and at the discretion of the local investigator. Hypertension should be treated aiming at a blood pressure < 140/90 mmHg. Fluid retention should be treated with diuretics, sodium- and fluid restriction or dialysis as required [45]. Diabetes mellitus is diagnosed and treated according to standard guidelines and at the discretion of the local investigator [46]. Dialysis is initiated and performed based on the discretion of the local investigator [45].

### Endpoints

#### Primary

- eGFR 3 months after inclusion (eGFR for patients on dialysis at 3 months is defined as “0 ml/min/1.73 m^2^).

#### Secondary

Renal function and changes in this defined by:

- Plasma creatinine (3 and 12 months)
- eGFR (12 months)
- Change in plasma creatinine at 3 and 12 months from the following time-points
  - ○ Highest measured plasma creatinine during the cause of AIN
  - ○ Plasma creatinine at time of renal biopsy
  - ○ Plasma creatinine at day 0 (inclusion)
- Number of patients with end stage renal failure and RRT (chronic dialysis or transplantation)
- For patients in whom a prior, baseline p-creatinine is available, the following will be analyzed
  - ○ Number of patients not on renal replacement therapy who returns to baseline creatinine or lower within 3 and 12 months after inclusion.
  - ○ Difference between p-creatinine after 3 and 12 months and baseline.
  Baseline p-creatinine is defined as the last recorded p-creatinine while the patient’s condition is considered as stable. If more than one such measurement is recorded within the last year, the baseline value is calculated as the average of the last 3 measurements. If more than one measurement is present from the same day an average of these measurements will be included as one of the 3 last measurements.
- In the prednisolone treated group the importance of “treatment delay” is analyzed by examining eGFR at 3 and 12 months. This will be explored using three different definitions of treatment delay:
  - ○ Days from first symptom to inclusion in this project and initiation of prednisolone
  - ○ Days from first contact to the Danish health care system to inclusion in this project
  - ○ Days from first contact to a nephrology department to inclusion in this project
- Quality of life estimated by a questionnaire (SF 36) at and 12 months

##### Additional exploratory analysis

The exploratory analyses include analysis of biomarkers in urine and plasma and the evaluation of these biomarkers in relation to renal prognosis. Renal biopsies form the included patients will be systematically re-evaluated to identify possible renal prognostic factors. The re-evaluation will include the presence and quantification of eosinophilic granulocytes, degrees of inflammation and fibrosis and additional staining for special biomarkers such as TGF-β1 and BMP-7. Safety end-point such as development of diabetes mellitus, infection rate, hospital admission and death will be explored.

## Methods

The local investigators are responsible for patient recruitment, clinical follow up, data entry in Redcap and shipment of centrally analyzed biochemical samples (biomarkers).

Blood and urine from biochemistry A, biochemistry B, 24-h urine collection and spot urine A, are analyzed at the local clinical biochemistry laboratory using standardized automated procedures. The CKD-EPI formula is used to calculate eGFR [47]. Similar, standardized and national reference intervals are applied by all clinical biochemistry laboratories in Denmark.

Blood sample C and spot urine B are analyzed at The University Clinic of Nephrology and Hypertension, Gødstrup Hospital using commercially available assays based on ELISA or RIA methods. Samples are centrifuged and frozen locally at − 80 °C, shipped to the central lab and stored in a research biobank at − 80 °C, until analyzed. Supplementary analyses of the material not described in the protocol is subject to additional approval by the scientific ethics committee.

Quality of life is estimated with the SF-36 questionary which the patients fill in 4 times during the study period [48, 49].

Safety estimates are evaluated in several ways. This includes the frequency of side effects, adverse events and hospital admissions, which are reported continuously by treating physicians in the eCRF. Development of diabetes in estimated by momentary and long term blood glucose levels (Hemoglobin A1c) and new onset hypertension from the blood pressure levels and medical chart reported in eCRF.

### Pathology

Renal biopsies forming the basis for the diagnosis and patient inclusion are performed at the local pathology services. The histological diagnosis of AIN is based on the finding of inflammatory cells in the renal tissue, primarily in the interstitium and possibly in the renal tubules, while blood vessels and glomeruli are spared [7, 15, 18].

Subsequently, all biopsies are evaluated and scored by the Dept. of Pathology, Odense University Hospital. A complete analysis plan is under development, but the evaluation should involve the staining for inflammation, fibrosis and tubular damage markers, including TGF-β1, BMP-7, A1M, B2M, MCP-1, NGAL, NAG, IL-18, TIMP-2, KIM-1 and IGFBP7.

### Statistics

The primary analysis will be performed as “intention to treat”, and the secondary analysis as “treated as”. Normally distributed variables are compared by unpaired t-test or one-way ANOVA. Repeated measures are analyzed using a general linear model (GLM). Comparable non-parametric tests are used in case of non-normally distributed variables. Both absolute and relative proportions are analyzed. Secondary end-points are analyzed as differences in absolute or relative proportions. Analysis of factors affecting the primary end-point will be performed using a GLM. Kaplan Meier curves will be used when appropriate e.g. in the evaluation of time to end stage renal failure. Any correlation between the primary end point and relevant secondary outcomes and outcomes form the exploratory analysis will be evaluated with appropriate adjustment for potential confounders.

Sample size was estimated for the primary outcome based on a clinical relevant 10 ml/min/1.73m^2^ difference in renal function (eGFR, CKD-EPI) between groups at 3 months, a standard deviation of 18 ml/min, a power of 0.80 and a significance level of 0.05. This showed that 51 patients should be included in each group. Anticipating dropouts, we estimate that 55 patients in each group are necessary and plan to randomize 110 patients.

### Ethics

The study is conducted according to The Declaration of Helsinki and Good Clinical Practice guidelines and is approved by the local ethics committee (Case number: 1–10-72-1008-17) and Danish Health Authorities (EudraCT number: 2017–000992-10, case number 2017043424). The study is monitored by the Good clinical practice (GCP) unit at Aalborg and Aarhus University Hospitals.

All patients with a biopsy with AIN will be approached by a physician and will be given oral information as well as a detailed written information. Provided the patients feel they have had sufficient time to consider their potential involvement, consent may be sought immediately. Otherwise, time is allowed for potential participants to consider the information provided, discuss the trial with their family and friends, and decide whether to take part before consenting. Informed written consent is obtained from all patients who agree to enter the study.

### Data protection

All data activities in the study are documented and stored in the web-based REDCap data capture application (https://redcap.au.dk) and is administered by Aarhus University. This system is situated in a Server Park in Central Denmark Region using firewall and Threat Management Gateway. Backup of data is performed on weekly basis and data transactions fulfil the requirements requested by the Danish Data Protection Agency.

### Time schedule

The estimated trial duration is 8 years. The study was launched at the first centers on Sept 1, 2017. Since then, all 11 Danish nephrology centers have been enrolled caring for a population of approximately 4.7 million adults. Currently, 30 patients have been included, and 82 patients have been screened. Unpublished data suggest that approximately 15 patients per year is diagnosed with AIN in the Middle- and Southern regions of Denmark covering approximately 2.0 million adults. With the participation from all Danish nephrology centers, we expect recruitment to be feasible within the suggested time-frame.

## Discussion

The study will provide important information on the effects of prednisolone treatment in AIN and as well as prognostic information relevant for future use of biomarkers and histology. Ultimately, this would lead to improved and evidence based clinical guidelines for the treatment of AIN. For years, treatment of AIN has been empirical, steered by clinical judgement and influenced by the results of retrospective studies hampered by selection bias. Many patients receive steroid treatment despite the lack of convincing evidence from randomized clinical trials. Treatment is driven by the potential long term benefits associated with the conservation of renal function, as chronic kidney disease has serious detrimental effects on morbidity and life expectancy. However, considering the potential serious adverse effects associated with steroids, the benefits of treatment should be better documented.

The prospective design with randomization to treatment should minimize selection bias for the patients who accept to participate. However, a selection prior or during recruitment is still a risk in this randomized design. Preferences by treating physicians may still determine and affect eligibility for participation in the trial. Thus, a systematic screening log of patient with renal biopsy proven AIN has been included allowing further characterization of the patients not included in the trial. This should enable us to identify if particular patient groups are withheld participation in the study. The study is a national Danish study and the Danish population is mainly of a Caucasian race and will therefor probably mainly include a Caucasian population. This will result in a uniform cohort but limit the generalizability to other populations with different racial composition.

Although we have no data to support this, it is likely that the threshold for renal biopsy in suspected AIN differs between centers depending on local practices, tradition and logistics. A direct consequence of this study may be that biopsy frequency increases to facilitate study participation. The requirement for a renal biopsy ensures the diagnosis of AIN and randomization minimize the risk of confounding due to different biopsy practices.

The open-label design may introduce bias both with respect to in the adjunctive therapy as well as in the reporting of outcomes both from participants and from the treating physicians. Additional treatment, including renal replacement therapy is decided by the treating physicians; however, is expected to follow international guidelines [45, 46, 50], Blinding was considered, but was discarded since a genuine blinding of high dose prednisolone treatment is difficult based on the significant adverse effects, including prominent leukocytosis. An inevitable and direct consequence of the open-label design is a risk of cross-over between treatment groups e.g. due to preferences of patients or treating physicians. To accommodate this, both “intention-to-treat” and “treated-as” analyses are planned. Nevertheless, a high cross over rate could influence and dilute a possible significant result, which is difficult to overcome statistically.

Although steroid treatment is often used in AIN there is limited evidence from clinical or retrospective trials to support this. Thus, considering the known and potential serious adverse effect of steroids, we believe that it is medically and ethically justified to include a trial arm that do not receive active treatment. Notably, recent studies, such as the PEXIVAS trial, addressing autoimmune disease in which the efficacy of steroid treatment seems evident, have shown that a reduction in steroid dose is safe and reduces adverse events [51].

The power calculations are based on a clinical relevant difference in eGFR of 10 ml/min/1.73m^2^ as well as additional assumptions based on previous observations from retrospective trials [29–31]. As in other trials there is a risk that the assumptions are wrong and the study is underpowered, in particular if there is significant drop out or cross-over. It may be argued that a difference in eGFR less than 10 ml/min/1.73m^2^ at 3 months could be clinical relevant, in particular in late stage CKD. Thus, a negative outcome of the trial cannot exclude a minor and possible relevant effect of prednisolone treatment in AIN; however, based on the incidence of AIN we believe that a larger trial was not feasible. Thus, even if the trial does show a significant effect of prednisolone in AIN, such potential minor effect may still be discussed with patients based on the individual case.

## Data Availability

Only the investigators will have access to the data. The datasets generated and analyzed during the current study are not publicly available but are available from the corresponding author on reasonable request. A dissemination plan has been developed which is directed towards different stakeholders a) Patients with chronic diseases, b) Health professionals, c) Politicians, d) Patient organisations, e) The general public, f) Scientific circles.

## Abbreviations

A1M: Alfa-1-mikrogolbulin
AIN: Acute interstitial nephritis
B2M: Beta-2-makroglobulin
BMP-7: Bone morphogenetic protein-7
eGFR: Estimated glomerular filtration rate
eCRF: Electronic Case report form
GLM: General linear model
IGFBP7: Insulin-like growth factor-binding protein 7
IL-18: Interleukin 18
KIM-1: Kidney injury marker 1
MCP-1: Monocyte chemotactic peptide-1
NAG: N-acetyl-beta-D-glucosaminidase
NGAL: Neutrophil gelatinase-associated lipocalin
PTH: Parathyroid hormone
TIMP-2: Tissue inhibitor of metalloproteinases-2
TGF-β1: Transforming growth factor-β1
